# Neonicotinoid exposure increases *Varroa destructor (Mesostigmata: Varroidae)* mite parasitism severity in honey bee colonies and is not mitigated by increased colony genetic diversity

**DOI:** 10.1093/jisesa/ieae056

**Published:** 2024-05-28

**Authors:** Lewis J Bartlett, Suleyman Alparslan, Selina Bruckner, Deborah A Delaney, John F Menz, Geoffrey R Williams, Keith S Delaplane

**Affiliations:** Center for the Ecology of Infectious Diseases, Odum School of Ecology, University of Georgia, Athens, GA 30602, USA; Department of Entomology, University of Georgia, Athens, GA 30602, USA; Department of Entomology & Plant Pathology, Auburn University, Auburn, AL 36849, USA; Department of Entomology & Plant Pathology, Auburn University, Auburn, AL 36849, USA; Department of Entomology & Wildlife Ecology, University of Delaware, Newark, DE 27695-7613, USA; Department of Entomology & Wildlife Ecology, University of Delaware, Newark, DE 27695-7613, USA; Department of Entomology & Plant Pathology, Auburn University, Auburn, AL 36849, USA; Department of Entomology, University of Georgia, Athens, GA 30602, USA

**Keywords:** neonicotinoid, pesticide, parasite, genetic diversity, pollinator

## Abstract

Agrochemical exposure is a major contributor to ecological declines worldwide, including the loss of crucial pollinator species. In addition to direct toxicity, field-relevant doses of pesticides can increase species’ vulnerabilities to other stressors, including parasites. Experimental field demonstrations of potential interactive effects of pesticides and additional stressors are rare, as are tests of mechanisms via which pollinators tolerate pesticides. Here, we controlled honey bee colony exposure to field-relevant concentrations of 2 neonicotinoid insecticides (clothianidin and thiamethoxam) in pollen and simultaneously manipulated intracolony genetic heterogeneity. We showed that exposure increased rates of *Varroa destructor* (Anderson and Trueman) parasitism and that while increased genetic heterogeneity overall improved survivability, it did not reduce the negative effect size of neonicotinoid exposure. This study is, to our knowledge, the first experimental field demonstration of how neonicotinoid exposure can increase *V. destructor* populations in honey bees and also demonstrates that colony genetic diversity cannot mitigate the effects of neonicotinoid pesticides.

## Introduction

Bees are critical to agricultural resilience by providing pollination services for a swathe of speciality crops (Klein et al. 2007, Potts et al. 2016, Knapp et al. 2017, Delaplane 2021). However, both wild and managed bees are threatened by interacting combinations of stressors, including forage loss, parasite pressures, and exposure to agrochemicals, including pesticides (Genersch et al. 2010, Pettis and Delaplane 2010, Potts et al. 2010, vanEngelsdorp and Meixner 2010, Goulson et al. 2015, Manley et al. 2015, Bird et al. 2021). Agricultural pesticides are known to interact in their toxicity, leading to suspicions that their dangers to bee pollinators are underestimated (Siviter et al. 2021). A wealth of literature has demonstrated the particularly pernicious threat of neonicotinoid use in agriculture in compromising bee health (see recent reviews by Lundin et al. (2015) and Singla et al. (2021)), leading to demand for better understanding of how to protect agricultural pollinators from pesticide exposure.

With managed western honey bees (*Apis mellifera* L.), beekeepers can make numerous interventions to help protect their bees’ nutritional or parasite stressors; for example, supplementary feeding with proteinaceous or caloric feed substitutes, namely pollen patties and sugar syrup (Caron and Connor 2013, Sammataro and Weiss 2013, Gemeda et al. 2018, Mortensen et al. 2019), or treatment with parasiticides such as amitraz or formic acid (Roth et al. 2020) to control major parasites of concern, for example, *Varroa destructor* mites (Traynor et al. 2020, Jack and Ellis 2021). Further, cultural and biological management such as brood breaks, requeening, and timely colony splitting are used to greatly improve the vitality of honey bee colonies both for nutritional and parasite regulation (Jack and Ellis 2021). However, pesticide exposure remains an Achilles’ heel of beekeeper management, with very few management tools available to beekeepers for mitigating its impact.

A core contributor to honey bee colony function and vitality is genetic diversity as a consequence of honey bee queen polyandry. Western honey bee queens accrue enough sperm for their lifetime’s cohort of daughter workers after mating with only one drone (Rinderer et al. 1985), but they almost always exceed this, with average mating numbers of 12 males (Tarpy et al. 2004) (or higher, see Withrow and Tarpy 2018), with significant variation in the number of matings including frequent cases much higher than this (Estoup et al. 1994). Multiple mechanisms have been proposed and evidenced to explain why increased polyandry improves colony function, health, and fitness. While it is widely understood that genetic diversity helps protect populations from infectious disease outbreaks (Ekroth et al. 2019, Gibson and Nguyen 2021), most of the importance of genetic diversity in honey bees is attributable to task thresholding, differentiation, and specialism (Delaplane et al. 2021, 2024).

Allelic richness amongst workers increases colony stability by diversifying task response thresholds so that environmental stimuli do not cause all worker cohorts to switch tasks at the same time (Oldroyd and Thompson 2006). For example, variation in stimulus thresholds that trigger the switch to fanning behavior ensures that as colony temperatures rise, the number of fanners increases sequentially such that only the minimum number of fanners necessary to cool the colony are engaged at any time (Calderone et al. 1989, Calderone and Page 1996, Eckholm et al. 2015). Similar principles apply to variation in the ability to forage on certain flowers, the tendency to prioritize pollen vs nectar or propolis foraging, and hygienic behaviors (to name a few behavioral phenotypes).

Further, it is hypothesized that certain patrilines are genetically predisposed to certain tasks by being hyper-sensitive to focal stimuli, which, when coupled with innate honey bee learning tendencies, enable individuals to become specialists. Hygienic behaviors whereby certain patrilines act as “colony leukocytes” due to their hypersensitivity to removing infected or sick brood, including policing *Varroa* parasitism (Boecking et al. 2000, Mondet et al. 2020, van Alphen and Fernhout 2020) are a demonstration of this principle. Correspondingly, increasing colony genetic diversity via higher polyandry has demonstrable positive effects on colony health. For example, a wealth of studies have linked increased queen polyandry to reduced parasite (including *Varroa*) pressure in colonies (Sherman et al. 1988, Shykoff and Schmid-Hempel 1991b,a, Tarpy and Seeley 2006, Seeley and Tarpy 2007, Delaplane et al. 2015, 2024, Desai and Currie 2015) as well as improved pesticide resilience, improved foraging rates, and greater honey production (Crozier and Page 1985, Oldroyd et al. 1992, Fewell and Page 1993, Dreller et al. 1995, Mattila and Seeley 2007, Girard et al. 2011, Rangel et al. 2013).

Given the ubiquitous importance of polyandry for colony vitality and the acute pressure placed on honey bees by pesticides, we sought to examine whether manipulation of colony genetic diversity via a management intervention (brood transplantation) could offset or even reduce the impact of a pesticide stressor on experimental honey bee colonies. Brood transplantation is a common beekeeping practice whereby capped brood are moved from one colony to another, typically to strengthen weak colonies. A collateral effect of this manipulation is a temporary increase in genetic diversity by introducing bees of novel genotype (and presumably phenotype) to the recipient colony.

We used brood exchange (transplantation) treatments to simulate the effects of a higher degree of polyandry in a colony. We tested whether this induced period of genetic heterogeneity not only improved colony health (for example, better brood survivorship, greater pollen foraging, reduced parasitic *Varroa* pressure, and increased queen survival) but also whether it improved colony tolerance to exposure of a mix of 2 common agricultural neonicotinoids (clothianidin and thiamethoxam) fed to colonies in contaminated pollen. The putative effect drivers are diversity-based improvements in pesticide detoxification coupled with improved colony tolerance to additive stressors. Our working hypotheses were: (i) that colonies fed neonicotinoid-dosed pollen would exhibit weaker indicators of vitality compared to control colonies; (ii) that colonies, where brood was mixed (exchanged between colonies), would exhibit stronger indications of vitality compared to control colonies; and finally, (iii) that these 2 treatments would interact such that the negative effects of neonicotinoid exposure were smaller in magnitude in those colonies in which brood was mixed.

## Materials and Methods

### Experimental Setup and Design

**Fig. 1.**
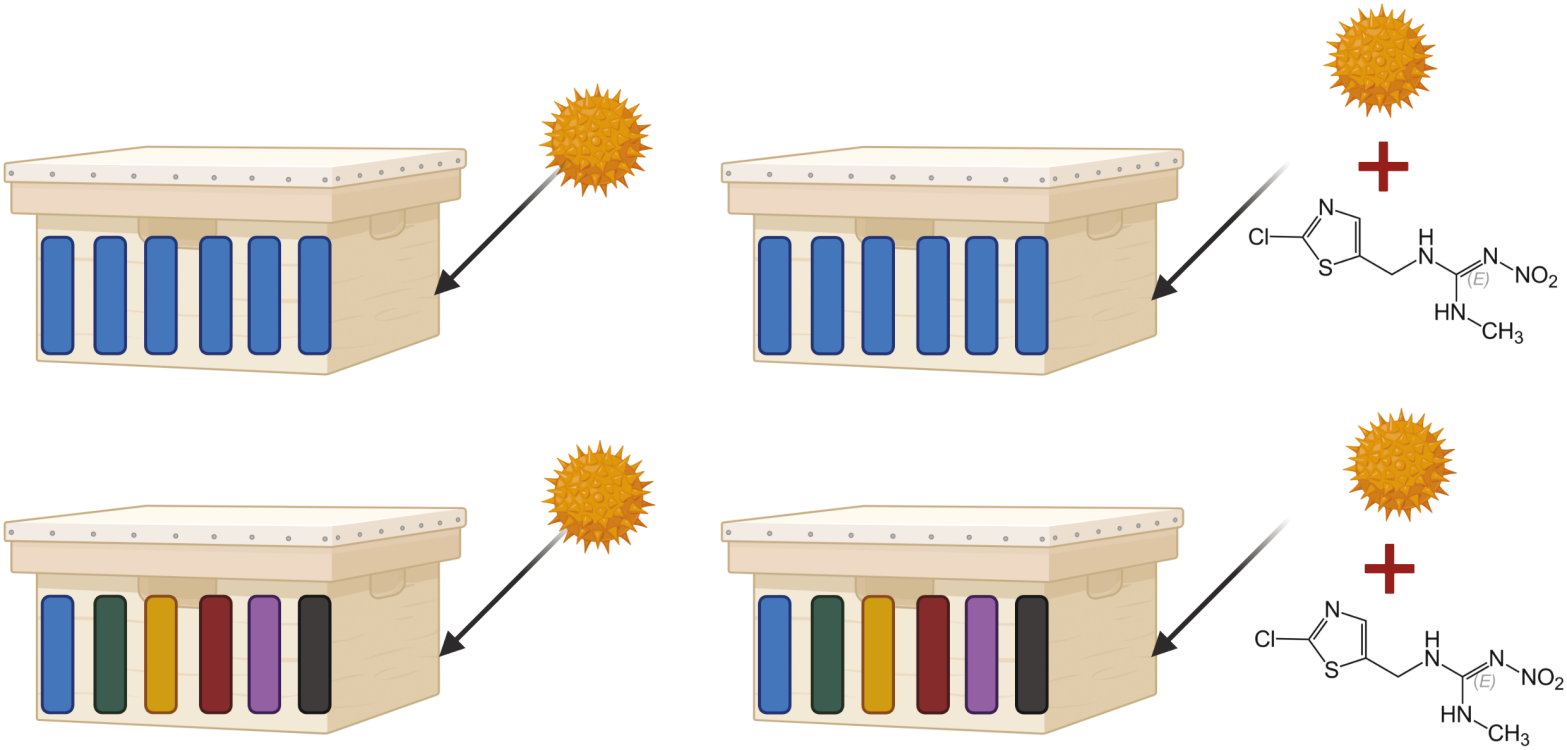
Single-colony examples of the 4 treatments. Top-row colonies are unmixed: all 6 brood frames come from the 1 resident queen mother; bottom-row colonies are mixed: each of the 6 brood frames comes from a different donor mother/colony (denoted by color). All colonies are fed pollen; however, right-column colonies are fed pollen dosed with 2 added neonicotinoids (here, denoted by clothianidin). Due to variation in colony strength across sites, only Delaware used colonies with 6 brood frames (sextets); Alabama and Georgia used colonies with 3 brood frames (triplets). Figure made in *Biorender* ©.

We conducted mirrored (but not simultaneous) experiments across 3 sites; research apiaries were located at the University of Delaware in Delaware, USA; University of Georgia in Athens, GA, USA; and Auburn University in Alabama, USA. Each site had a single apiary of 24 experimental colonies of managed western honey bees, split evenly across a 2 × 2 treatment plan (6 colonies per unique treatment combination). Treatments were either brood-mixed or unmixed, and colonies were given either “clean” pollen patties or pollen patties dosed with field-relevant neonicotinoid pesticides (see Fig. 1). Queens were purchased open-mated from a single supplier (Rossman Apiaries, USA) and inserted initially into 5-frame nucleus colonies of empty drawn comb and given time to establish until transplantation into typical 10-frame Langstroth hives before colonies grew until the minimum number of brood frames necessary for the experiment to begin were available (2–4 months across sites). We allowed colonies to exhibit their typical natural variation in size and did not standardize beyond the initial nucleus colony establishment.

Experimental “day 0” was the day that brood mixing occurred in colonies assigned to those treatment groups, whereby brood frames were exchanged among colonies previously assigned to triplets or sextets depending on colony strength and available brood across sites (Fig. 1). Where 6 frames of brood were reliably available in all colonies, sextets were used. In other regions, where colonies only reached 3 frames of brood, triplets were used. In each triplet or sextet, each member colony retained one frame of brood from its resident mother queen and donated (and reciprocally received) one frame of brood from each other member of the group (such that where colonies were in sextets, mixed colonies donated 5 frames of brood and received 5 “foreign” brood frames, while colonies in triplets received 2 “foreign” brood frames to replace the 2 they donated). All colonies (mixed and unmixed) were fully assessed at this stage, including quantifying brood area and discriminating brood into eggs, uncapped, or capped; we refer to this as the “experimental brood cohort.” Pollen patties were made in Alabama (Auburn Bee Lab) using corbicular pollen (60% w/w) sourced from Colorado, powdered sugar (30% w/w, food-grade cane sucrose), and organic honey (10% w/w). Corbicular pollen was tested for agricultural chemical residues using liquid chromatography-tandem mass spectrometry (Mullin et al. 2010) and showed no residues above detection limits. “Clean” pollen patties were unaltered from the above. Neonicotinoid-dosed patties were supplemented with 4.5 ng g^−1^ thiamethoxam (Sigma-Aldrich) and 1.5 ng g^−1^ clothianidin (Sigma-Aldrich) at the mixing stage. These concentrations reflect those found in pollen from flowering crops and crop-adjacent wildflowers in agricultural systems using these neonicotinoids (David et al. 2016). While “field-realistic” doses have a disputed history (Carreck and Ratnieks 2014), a recent study by Graham et al. (2021) looking specifically at corbicular pollen of honey bees in both managed and unmanaged fields found concentrations at or above the ones we use here. Mixtures were compressed into ~225 g patties used for feeding. Patties were placed in the corresponding colonies on experimental day 7 and replaced throughout the experiment to allow ad libitum feeding. Colonies were forced to consume their pollen patties by placing pollen traps on all colony entrances to deny the importation of foraged pollen. Force-feeding of pollen lasted 30 days, and visual inspection confirmed pollen patty consumption. Overall, this approach to achieving field-realistic forced-feeding of neonicotinoid-dosed pollen precisely mirrors numerous other studies to better allow comparison of results between studies using this model exposure scenario (e.g., Straub et al. 2016, 2019, 2021, Forfert et al. 2017, Friedli et al. 2020, Bruckner et al. 2021). Colony monitoring proceeded throughout the experiment as described below. We note that the main experimental periods were asynchronous across sites based on colony establishment dates, availability of personnel, and season length.

### Colony Monitoring and Data Collection

We measured up to 8 main colony response variables in all apiaries at all sites. These were size (capped brood area), queen survival (whether marked queens were confirmed as still present), pollen (mass of corbicular pollen gathered in 24 h), aggression (recruitment of guard bees to colony entrance following alarm pheromone exposure), per-capita *Varroa* parasitism (phoretic mites washed using ethanol from 300 adult bees), *Varroa* mite drop (mite drop rate onto sticky screen colony bottom boards), comb construction (area of comb constructed onto undrawn frame inserted for 24 h), and brood survival (proportion of identified L1–L2 larvae that survived to capping). All 8 response variables were recorded for the Delaware sites; Georgia sites could not confirm queen survival or undertake alcohol washes for per-capita *Varroa* loads, and Alabama sites could not confirm queen survival, measure pollen foraging rate, or measure comb construction.

*Size* was assessed by 2 independent observers in the style of Delaplane et al. (2013), whereby each assessor estimated the proportion of each frame side in a given colony covered by capped brood, yielding a response variable measured in “Langstroth deep frames of capped brood”; each site measured this size variable multiple times. *Queen survival* was recorded at the end of the experiment and is a binary response variable showing whether a given colony had the same original marked queen as introduced at the start of the experiment. *Pollen* was measured as the mass in grams of corbicular pollen collected in the pollen trap placed on the entrance of each colony over a 24-h period and was collected throughout the experiment. *Aggression* was assayed by counting the number of bees visible on the landing board outside a colony, then placing a cotton ball soaked with 50 µl of isoamyl acetate (Acros Organics, USA) onto the landing board for 120 s, and then recording the number of bees again on the landing board of the colony to estimate recruitment in response to alarm pheromone. *Mite parasitism* was estimated for each colony by collecting 300 adult honey bees from the surface of brood frames, submerging them in 70% ethanol, straining them with Varroa EasyCheck washers (Véto-pharma, France), and counting the number of mites dislodged and removed; this wash and count procedure was repeated until 2 consecutive “zero” counts were observed. *Mite drop* was estimated by placing sticky screen bottom boards (Mann Lake, USA) on the bottom of each colony for 72 h and then counting the number of *Varroa* mites which had fallen and adhered onto each screen surface. *Comb construction* was assessed by inserting a frame of undrawn comb with wax foundation into the colony for 24 h; the proportion of the frame fully drawn with comb after this period was then measured. Finally, *brood survival* was repeatedly assessed for each colony by identifying a minimum of 20 cells containing larvae at their first or second instars and marking the cell locations on an acetate “coverslip” that can be held over the brood frame; these cells were then monitored to see if the young larvae survived to the capping stage or were removed (either following death or infanticide). These larvae were not transplants and were young enough to be the guaranteed progeny of the queen in that colony.

Timing of measurements for each response variable was guided on a site-specific basis by the stochastic computational model of Bartlett et al. (2021b), which combines published schedules of temporal task allocation in honeybees (Seeley 1982) with the measured age distribution of brood we inserted into hives to track a polyandrous cohort as it ages through its expected succession of task peaks. For example, assessments of brood survivorship were undertaken shortly after the beginning of the experiment at each site, as we estimate the experimental brood cohort would be undertaking nursing behavior from between approximately 7–21 days postmix; in contrast, the same cohort’s contribution to the task of guarding the colony peaked at days 24–28 across sites, which is when aggression assays were targeted. We present full predictions of when the assessed cohorts were expected to contribute to different tasks in the supplementary material (Supplementary Figs. S1–S3).

### Statistical Analysis

All analyses were undertaken in the statistical programming language “R” (R Core Team 2019) version 4.0.2. All analyses used a linear modeling framework, where linear models (lm) were fit to data and then fixed predictor terms were tested for significance using type-III ANOVAs. Directions and sizes of significant effects were then taken from the corresponding fit model where found. In the single case of queen survival, which was only recorded at the Delaware site, we could fit a typical generalized linear model (glm) with a binomial error structure to the binary response variable (queen survived {0}, queen presumed dead {1}) with 2 interacting fixed predictors: whether a colony was mixed or not (0,1) and dosed with neonicotinoids or not (0,1)—see Fig. 1. We used the same fixed predictor structure for all other response variable analyses. However, for all other response variables, we included side effects by using mixed modeling ((g)lmms) approach to account for the nested (hierarchical) nature of our data, with individual colonies often sampled multiple times and only ever belonging to one site; we fit these (g)lmms and tested for significance using the “*afex*” package (Singmann et al. 2019), which wraps around the “*lme4*” package (Bates et al. 2015). Model type and error structure depended on the nature of the response variable; some variables were suitable for analysis assuming a Gaussian (normal) error structure and, therefore, appropriate for linear mixed modeling. Other variables required generalized linear mixed models with binomial- or Poisson-distributed error structures. Test statistics used for the type-III ANOVAs varied accordingly (F-statistic for linear mixed models, Chi-squared for generalized). In all cases, the fixed predictors were as above (Mix + Neonic + Mix:Neonic). In the case of aggression, we added an additional fixed predictor term to account for the number of bees present at the entrance prior to pheromone application. We make all analysis and data available as a Zenodo-archived GitHub repository (10.5281/zenodo.10899691).

## Results

**Fig. 2.**
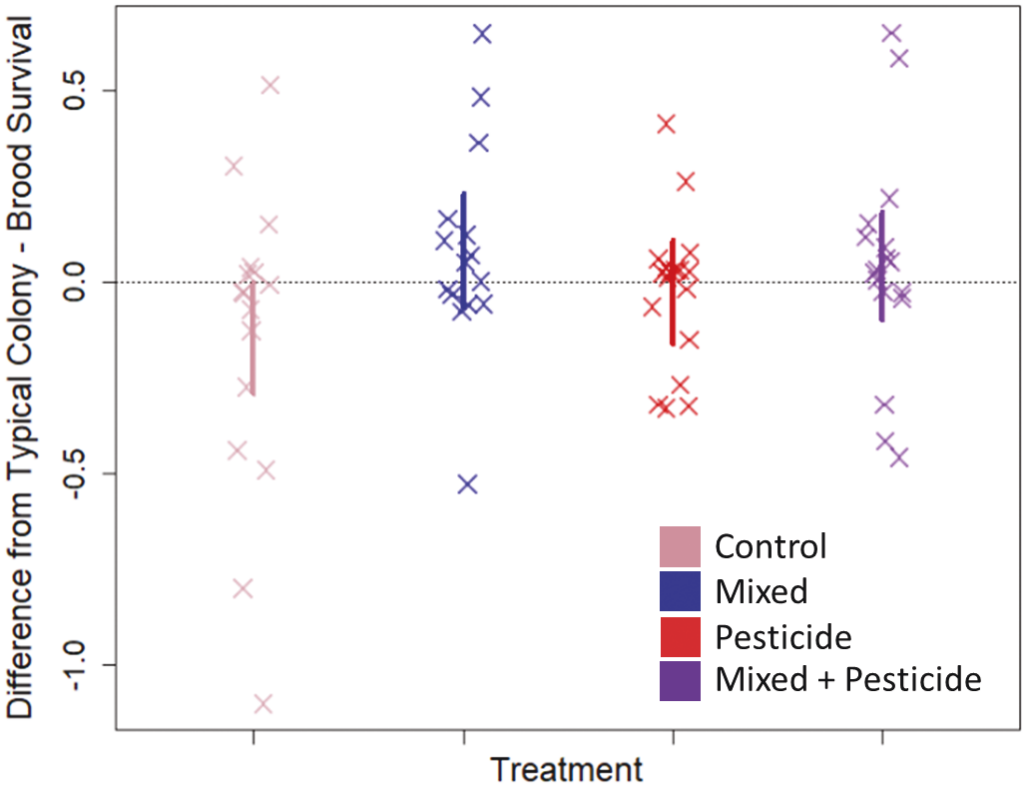
Summary graph of survival of honey bee larvae across the experiment. Each point represents 1 colony, grouped according to treatment. Values for each colony are summary measures calculated using scaled residuals for each location × day block and are for illustrative purposes: plotted lines are naïve 95% CIs and do not directly correspond to statistical results—they are plotted for assistance in interpreting graphical results only. We found a significant positive effect of brood mixing on larval survival rates.

**Fig. 3.**
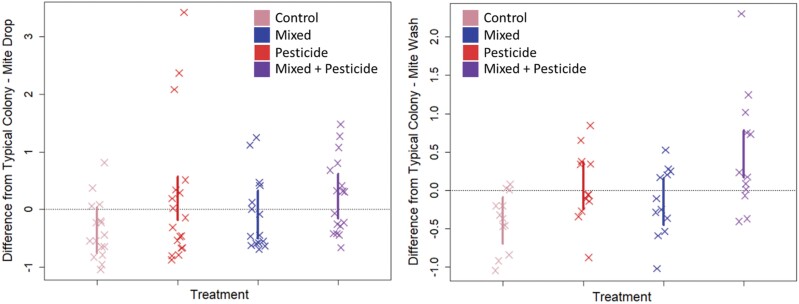
Summary graphs of mite sticky screen drop rates (panel A, left) and per-capita (mites per 100 bees) phoretic mite counts from ethanol washes (panel B, right). Each point represents 1 colony, grouped according to treatment. Values for each colony are summary measures calculated using scaled residuals for each location and day block. Plotted lines are naïve 95% CIs and do not directly correspond to statistical results—they are plotted for assistance in interpreting graphical results only. In both instances, we found increasing *Varroa* mite load in the presence of neonicotinoid exposure.

We summarize the type-III ANOVA results run on our (g)lm(m)s in Table 1. We find no evidence of an interaction effect between neonicotinoid exposure and brood exchange in any instance. However, we do find some singular effects of each on specific response variables. Notably, we find evidence of a positive effect of brood exchange (mixing) on subsequent larval survivorship (*χ*^2^_1,7_ = 3.93, *P* = 0.05) where larvae at the L1–L2 stage (1st–2nd instar) in mixed colonies showed survival rates on average 1.74× higher (1.01–2.98 × 95% CI) than those in corresponding unmixed colonies (Fig. 2). We also find evidence of a detrimental effect of neonicotinoid exposure on colonies, where both mite drop rates (*χ*^2^_1,7_ = 3.91, *P* = 0.05) and mite wash counts (*χ*^2^_1,7_ = 8.04, *P* = 0.005) were higher in colonies exposed to neonicotinoids; colonies exposed to neonicotinoids had on average 5.3 (2.3–9.3 95% CI) more mites drop in 24 h and 1.11 (0.67–1.61 95% CI) more phoretic mites per 100 bees (see Fig. 3). We found no evidence of treatment effects on aggression, comb construction, pollen gathering rates, queen survival, or brood area.

**Table 1.** Summary of statistical testing results. Bolded lines highlight significant tests of biological importance. Model type includes the testing specification of degrees of freedom

Response variable	Fixed predictor	Test	*P*-value	Model type
Aggression				lmm (KR)
	EntranceBees (Start)	*F*_1,238_ = 39.26	<0.001	
	Neonicotinoid	*F*_1,48_ = 2.89	0.10	
	Mixed	*F*_1,49_ = 0.37	0.55	
	Neonicotinoid: Mixed	*F*_1,48_ = 0.90	0.35	
**Brood survival**				binomial glmm (LRT)
	Neonicotinoid	χ^2^_1,7_ = 1.76	0.18	
	**Mixed**	**χ** ^**2**^_**1,7**_ ** = 3.93**	**0.05**	
	Neonicotinoid: Mixed	χ^2^_1,7_ = 0.63	0.43	
Comb construction				Poisson glmm (LRT)
	Neonicotinoid	χ^2^_1,7_ = 0.15	0.09	
	Mixed	χ^2^_1,7_ = 0.92	0.34	
	Neonicotinoid: Mixed	χ^2^_1,7_ = 1.28	0.26	
**Mite drop**				Poisson glmm (LRT)
	**Neonicotinoid**	**χ** ^**2**^_**1,7**_ ** = 3.91**	**0.05**	
	Mixed	χ^2^_1,7_ = 1.46	0.24	
	Neonicotinoid: Mixed	χ^2^_1,7_ = 0.33	0.57	
**Mite wash**				Poisson glmm (LRT)
	**Neonicotinoid**	**χ** ^**2**^_**1,7**_ ** = 8.04**	**0.005**	
	Mixed	χ^2^_1,7_ = 0.01	0.41	
	Neonicotinoid: Mixed	χ^2^_1,7_ = 0.20	0.66	
Pollen gathering rate				lmm (KR)
	Neonicotinoid	*F*_1,38_ = 1.22	0.28	
	Mixed	*F*_1,37_ = 1.83	0.18	
	Neonicotinoid: Mixed	*F*_1,38_ = 1.54	0.22	
Brood area				lmm (KR)
	Neonicotinoid	*F*_1,37_ = 1.48	0.23	
	Mixed	*F*_1,37_ = 0.33	0.57	
	Neonicotinoid: Mixed	*F*_1,37_ = 0.10	0.76	
Queen survival				glm (Wald)
	Neonicotinoid	χ^2^_1,7_ = < 0.01	0.99	
	Mixed	χ^2^_1,7_ = 0.34	0.56	
	Neonicotinoid: Mixed	χ^2^_1,7_ = < 0.01	0.99	

## Discussion

We found that field-relevant neonicotinoid exposure increased the severity of *Varroa* parasitism rates in exposed colonies. We did not find evidence supporting the hypothesis that this negative effect could be mitigated by increased genetic diversity in the colony, as in no instance did we find a significant interaction between pesticide exposure and brood mixing. Additionally, we found no evidence that increased genetic diversity compensated for the negative effects of the neonicotinoids, as the 2 treatments did not overlap in which colony phenotypes they significantly affected. We did, however, confirm the beneficial effects of increased genetic diversity in the form of improved brood survival.

There is no shortage of observational and experimental studies emphasizing the synergistic impacts of pesticide and parasite exposure on honey bee health (Goulson et al. 2015, Zee et al. 2015, Dolezal et al. 2016, Sánchez-Bayo et al. 2016, Bartlett et al. 2021a), including neonicotinoid and *Varroa* pressure (Straub et al. 2019, Tesovnik et al. 2019, Morfin et al. 2020). Moreover, field observations have explicitly linked neonicotinoid exposure to vulnerability to *Varroa* (Alburaki et al. 2015, Dively et al. 2015), with crucial laboratory studies by Annoscia et al. (2020) demonstrating how the neonicotinoid clothianidin increases *Varroa* reproduction rates due to reducing honey bee hemolytic immune response. Our work completes this literature body by showing that the individual-level findings of Annoscia et al. (2020) are mirrored at the colony level with contaminated pollen (see Fig. 3) and is the first (to our knowledge) demonstration of this link between neonicotinoid and *Varroa* parasitism using a manipulated field experiment. We emphasize that neonicotinoid exposure increasing vulnerability to *Varroa* parasitism and subsequent increased mite abundance is a subtly different, possibly supplementary, mechanism to simply demonstrating whether the combination of pesticide exposure and parasitism has antagonistic, additive, or synergistic impacts on honeybee health compared to either effect in isolation—possibly explaining the mixed range of outcomes reported for these interactions (Straub et al. 2019, Harwood and Dolezal 2020, Bird et al. 2021, Bruckner et al. 2021, Siviter et al. 2021).

Substituting heightened polyandry by diversifying colony origins of brood through brood transplantation directly improved the survival of L1–L2 larvae to the capping stage, adding to a growing body of evidence supporting the colony-level benefits of polyandry. If one assumes that a queen mates with, and her progeny are sired by, an average of 12 males (Tarpy et al. 2004), then our colonies in Alabama and Georgia were made up of approximately 12 patrilines (unmixed) or temporarily 36 patrilines (mixed, triplets of 12 × 3); correspondingly, colonies in Delaware were of the order of 12 patrilines (unmixed) or temporarily 72 patrilines (mixed, hextets of 12 × 6). Delaplane et al. (2021) showed increased brood survival in colonies whose queens were instrumentally inseminated with 54 males compared to colonies whose queens were inseminated with 9 males. As mentioned above, degrees of polyandry spanning this range was very likely achieved in the present study, supporting that the positive effects on brood survival were an effect of induced polyandry. Given our use of open-mated, “practitioner realistic” queens, we, therefore, present initial evidence that brood mixing could be a beekeeper-friendly method of simulating the beneficial effects of high queen mating number for improving colony strength; however, the strength of benefit needs further interrogation given how few colony strength indicators responded to mixing in this study. We do not anticipate brood mixing to be a major route of pathogen transmission, per the epidemiology of Bartlett et al. (2019).

It is possible that our neonicotinoid treatments were too subtle to detect interactions between brood mixing (which was also temporary) and pesticides. While our neonicotinoid treatments were at field-realistic doses (David et al. 2016), honey bees may be more resilient in the face of these pesticides compared to other bees (although not necessarily other insects or pollinators) (Claudianos et al. 2006, Klein et al. 2017). It is plausible that more extreme but less “realistic” experimental doses do a better job bracketing the range of ecological possibilities.

On the other hand, our failure to detect interactions may be a true reflection of reality. Our design was sufficiently powerful to show the direct effects of both fixed predictors in the directions anticipated. If the absence of interactions is legitimate, this highlights the continued challenges facing beekeepers (and wild bees more so) by the very limited number of mechanisms available for mitigating damage caused by agricultural insecticides. If a facet of honey bee biology as fundamental and influential as colony genetic heterogeneity is not capable of mitigating or buffering the impacts of insecticide stressors, it is difficult to envisage what can be done on the side of beneficial pollinators to improve resilience to insecticides, leaving responsibility for action squarely on the side of those land managers and practitioners who control insecticide application and exposure pathways.

The phenomena we observe are also worth consideration in their own right. The absence of an effect of the neonicotinoids on larval survival may be a testament to the resilience of honey bees in protecting their larvae from agrochemicals via nurse-bee buffering as well as the high tolerance of honey bee brood to these pesticides (Grillone et al. 2017). This may be especially true at our field-relevant doses, although prior work has found links to reduced nursing effectiveness and larval development inhibition from similar neonicotinoid doses (Siefert et al. 2020). The increased survival rates of young brood when raised by a more genetically heterogeneous cohort are more unsurprising, however, given the well-evidenced hypotheses discussed earlier concerning task partitioning and colony efficiency when genetic diversity is increased, and is a finding that has been observed elsewhere in prior work (Delaplane et al. 2021).

The lack of an effect of the genetic diversity manipulation on *Varroa* is more surprising but may be due to the ephemeral effects of simulated polyandry compared to permanent heightened polyandry, as found elsewhere in other studies (Tarpy et al. 2013, Delaplane et al. 2015). The impact of the neonicotinoid in increasing *Varroa* populations both in absolute terms and in per-capita parasitism is in agreement with laboratory and observational studies (Alburaki et al. 2015, Dively et al. 2015, Annoscia et al. 2020) and with this present work has now been demonstrated in the field, contributing to a large body of literature on the multiple and interacting stressors on pollinators. It is a notable finding that we confirm in-field that neonicotinoids can exacerbate the abundance of the parasite *Varroa*, which is arguably the single most severe contributor to managed honey bee losses in the United States (Traynor et al. 2020).

## Supplementary Material

ieae056_suppl_Supplementary_Material

## Data Availability

We will make all data and analysis code available on a Zenodo-archived repository [10.5281/zenodo.10899691].

## References

[CIT0001] Alburaki M , Boutin S, Mercier P-L, Loublier Y, Chagnon M, Derome N. Neonicotinoid-coated *Zea mays* seeds indirectly affect honeybee performance and pathogen susceptibility in field trials. PLoS One. 2015:10(5):e0125790. 10.1371/journal.pone.0125790 25993642 PMC4436261

[CIT0002] Annoscia D , Di Prisco G, Becchimanzi A, Caprio E, Frizzera D, Linguadoca A, Nazzi F, Pennacchio F. Neonicotinoid clothianidin reduces honey bee immune response and contributes to *Varroa* mite proliferation. Nat Commun. 2020:11(1):5887. 10.1038/s41467-020-19715-8 33208729 PMC7675992

[CIT0003] Bartlett LJ , Boots M, Brosi BJ, de Roode JC, Delaplane KS, Hernandez CA, Wilfert L. Persistent effects of management history on honeybee colony virus abundances. J Invertebr Pathol. 2021a:179(2):107520. 10.1016/j.jip.2020.107520 33359478

[CIT0004] Bartlett LJ , Bruckner S, Delaney DA, Williams GR, Delaplane KS. A computational approach to tracking age-based task frequency distributions of *Apis mellifera* worker cohorts. J Apic Res. 2021b:61(1):147–150. 10.1080/00218839.2021.1909313

[CIT0005] Bartlett LJ , Rozins C, Brosi BJ, Delaplane KS, Roode J.C. de, White A, Wilfert L, Boots M. Industrial bees: the impact of apicultural intensification on local disease prevalence. J Appl Ecol. 2019:56(9):2195–2205. 31588148 10.1111/1365-2664.13461 PMC6771535

[CIT0006] Bates D , Maechler M, Bolker B, Walker S. Fitting linear mixed-effects models using lme4. J Stat Softw. 2015:67:1–48.

[CIT0007] Bird G , Wilson AE, Williams GR, Hardy NB. Parasites and pesticides act antagonistically on honey bee health. J Appl Ecol. 2021:58(5):997–1005. 10.1111/1365-2664.13811

[CIT0008] Boecking O , Bienefeld K, Drescher W. Heritability of the *Varroa*-specific hygienic behaviour in honey bees (Hymenoptera: Apidae). J Anim Breed Genet. 2000:117(6):417–424. 10.1046/j.1439-0388.2000.00271.x

[CIT0009] Bruckner S , Straub L, Neumann P, Williams GR. Synergistic and antagonistic interactions between *Varroa* destructor mites and neonicotinoid insecticides in male *Apis mellifera* honey bees. Front Ecol Evol. 2021:9:735.

[CIT0010] Calderone NW , Page RE. Temporal polyethism and behavioural canalization in the honey bee, *Apis mellifera*. Anim Behav. 1996:51(3):631–643.

[CIT0011] Calderone NW , Robinson GE, Page RE. Genetic structure and division of labor in honeybee societies. Experientia. 1989:45(8):765–767. 10.1007/bf01974583

[CIT0012] Caron DM , Connor LJ. Honey bee biology and beekeeping, revised edition. Revised ed. Kalamazoo (MI): Wicwas Press; 2013.

[CIT0013] Carreck NL , Ratnieks FLW. The dose makes the poison: have ‘field realistic’ rates of exposure of bees to neonicotinoid insecticides been overestimated in laboratory studies? J Apic Res. 2014:53(5):607–614. 10.3896/ibra.1.53.5.08

[CIT0014] Claudianos C , Ranson H, Johnson RM, Biswas S, Schuler MA, Berenbaum MR, Feyereisen R, Oakeshott JG. A deficit of detoxification enzymes: pesticide sensitivity and environmental response in the honeybee. Insect Mol Biol. 2006:15(5):615–636. 10.1111/j.1365-2583.2006.00672.x 17069637 PMC1761136

[CIT0015] Crozier RH , Page RE. On being the right size: male contributions and multiple mating in social Hymenoptera. Behav Ecol Sociobiol. 1985:18:105–115.

[CIT0016] David A , Botías C, Abdul-Sada A, Nicholls E, Rotheray EL, Hill EM, Goulson D. Widespread contamination of wildflower and bee-collected pollen with complex mixtures of neonicotinoids and fungicides commonly applied to crops. Environ Int. 2016:88(3):169–178. 10.1016/j.envint.2015.12.011 26760714

[CIT0017] Delaplane K , Hagan K, Vogel K, Bartlett L. Mechanisms for polyandry evolution in a complex social bee. Behav Ecol Sociobiol. 2024:78:39.

[CIT0018] Delaplane KS. Crop pollination by bees, volume 1: evolution, ecology, conservation, and management. Wallingford, Oxfordshire, UK: CABI; 2021.

[CIT0019] Delaplane KS , Given JK, Menz J, Delaney DA. Colony fitness increases in the honey bee at queen mating frequencies higher than genetic diversity asymptote. Behav Ecol Sociobiol. 2021:75:126.

[CIT0020] Delaplane KS , Pietravalle S, Brown MA, Budge GE. honey bee colonies headed by hyperpolyandrous queens have improved brood rearing efficiency and lower infestation rates of parasitic *Varroa* mites. PLoS One. 2015:10(12):e0142985. 10.1371/journal.pone.0142985 26691845 PMC4686211

[CIT0021] Delaplane KS , Van Der Steen J, Guzman-Novoa E. Standard methods for estimating strength parameters of *Apis mellifera* colonies. J Apic Res. 2013:52(1):1–12.

[CIT0022] Desai SD , Currie RW. Genetic diversity within honey bee colonies affects pathogen load and relative virus levels in honey bees, *Apis mellifera* L. Behav Ecol Sociobiol. 2015:69:1527–1541.

[CIT0023] Dively GP , Embrey MS, Kamel A, Hawthorne DJ, Pettis JS. Assessment of chronic sublethal effects of imidacloprid on honey bee colony health. PLoS One. 2015:10(3):e0118748. 10.1371/journal.pone.0118748 25786127 PMC4364903

[CIT0024] Dolezal AG , Carrillo-Tripp J, Miller WA, Bonning BC, Toth AL. Pollen contaminated with field-relevant levels of cyhalothrin affects honey bee survival, nutritional physiology, and pollen consumption behavior. J Econ Entomol. 2016:109(1):41–48. 10.1093/jee/tov301 26476556

[CIT0025] Dreller C , Fondrk MK, Page RE. Genetic variability affects the behavior of foragers in a feral honeybee colony. Naturwissenschaften. 1995:82(5):243–245. 10.1007/s001140050179

[CIT0026] Eckholm BJ , Huang MH, Anderson KE, Mott BM, DeGrandi-Hoffman G. Honey bee (*Apis mellifera*) intracolonial genetic diversity influences worker nutritional status. Apidologie. 2015:46(2):150–163. 10.1007/s13592-014-0311-4

[CIT0027] Ekroth AKE , Rafaluk-Mohr C, King KC. Host genetic diversity limits parasite success beyond agricultural systems: a meta-analysis. Proc Biol Sci. 2019:286(1911):20191811. 10.1098/rspb.2019.1811 31551053 PMC6774778

[CIT0028] Estoup A , Solignac M, Cornuet J. Precise assessment of the number of Patrilines and of genetic relatedness in honeybee colonies. Proc R Soc Lond Ser B Biol Sci. 1994:258(1351):1–7.

[CIT0029] Fewell JH , Page RE. Genotypic variation in foraging responses to environmental stimuli by honey bees, *Apis mellifera*. Experientia. 1993:49(12):1106–1112. 10.1007/bf01929923

[CIT0030] Forfert N , Troxler A, Retschnig G, Gauthier L, Straub L, Moritz RFA, Neumann P, Williams GR. Neonicotinoid pesticides can reduce honeybee colony genetic diversity. PLoS One. 2017:12(10):e0186109. 10.1371/journal.pone.0186109 29059234 PMC5653293

[CIT0031] Friedli A , Williams GR, Bruckner S, Neumann P, Straub L. The weakest link: haploid honey bees are more susceptible to neonicotinoid insecticides. Chemosphere. 2020:242(3):125145. 10.1016/j.chemosphere.2019.125145 31678852

[CIT0032] Gemeda TK , Li J, Luo S, Yang H, Jin T, Huang J, Wu J. Pollen trapping and sugar syrup feeding of honey bee (Hymenoptera: Apidae) enhance pollen collection of less preferred flowers. PLoS One. 2018:13(9):e0203648. 10.1371/journal.pone.0203648 30208089 PMC6135515

[CIT0033] Genersch E , Ohe W. von der, Kaatz H, Schroeder A, Otten C, Büchler R, Berg S, Ritter W, Mühlen W, Gisder S, Meixner M, et al. The German bee monitoring project: a long term study to understand periodically high winter losses of honey bee colonies. Apidologie. 2010:41(5):332–352.

[CIT0034] Gibson AK , Nguyen AE. Does genetic diversity protect host populations from parasites? A meta-analysis across natural and agricultural systems. Evol Lett. 2021:5(1):16–32. 10.1002/evl3.206 33552533 PMC7857278

[CIT0035] Girard MB , Mattila HR, Seeley TD. Recruitment-dance signals draw larger audiences when honey bee colonies have multiple patrilines. Insectes Soc. 2011:58(1):77–86. 10.1007/s00040-010-0118-x 21350596 PMC3028068

[CIT0036] Goulson D , Nicholls E, Botías C, Rotheray EL. Bee declines driven by combined stress from parasites, pesticides, and lack of flowers. Science. 2015:347(6229):1255957. 10.1126/science.1255957 25721506

[CIT0037] Graham KK , Milbrath MO, Zhang Y, Soehnlen A, Baert N, McArt S, Isaacs R. Identities, concentrations, and sources of pesticide exposure in pollen collected by managed bees during blueberry pollination. Sci Rep. 2021:11(1):16857. 10.1038/s41598-021-96249-z 34413379 PMC8377133

[CIT0038] Grillone G , Laurino D, Manino A, Porporato M. Toxicity of thiametoxam on in vitro reared honey bee brood. Apidologie. 2017:48(5):635–643. 10.1007/s13592-017-0506-6

[CIT0039] Harwood GP , Dolezal AG. Pesticide–virus interactions in honey bees: challenges and opportunities for understanding drivers of bee declines. Viruses. 2020:12(5):566. 10.3390/v12050566 32455815 PMC7291294

[CIT0040] Jack CJ , Ellis JD. Integrated pest management control of *Varroa destructor* (Acari: Varroidae), the most damaging pest of (*Apis mellifera* L. (Hymenoptera: Apidae)) colonies. J Insect Sci. 2021:21(5):6. 10.1093/jisesa/ieab058 PMC8449538 34536080

[CIT0041] Klein A-M , Vaissière BE, Cane JH, Steffan-Dewenter I, Cunningham SA, Kremen C, Tscharntke T. Importance of pollinators in changing landscapes for world crops. Proc Biol Sci. 2007:274(1608):303–313. 10.1098/rspb.2006.3721 17164193 PMC1702377

[CIT0042] Klein S , Cabirol A, Devaud J-M, Barron AB, Lihoreau M. Why bees are so vulnerable to environmental stressors. Trends Ecol Evol. 2017:32(4):268–278. 10.1016/j.tree.2016.12.009 28111032

[CIT0043] Knapp JL , Bartlett LJ, Osborne JL. Re-evaluating strategies for pollinator-dependent crops: How useful is parthenocarpy? J Appl Ecol. 2017:54(4):1171–1179. 10.1111/1365-2664.12813 28781379 PMC5516152

[CIT0044] Lundin O , Rundlöf M, Smith HG, Fries I, Bommarco R. Neonicotinoid insecticides and their impacts on bees: a systematic review of research approaches and identification of knowledge gaps. PLoS One. 2015:10(8):e0136928. 10.1371/journal.pone.0136928 26313444 PMC4552548

[CIT0045] Manley R , Boots M, Wilfert L. REVIEW: emerging viral disease risk to pollinating insects: ecological, evolutionary and anthropogenic factors. J Appl Ecol. 2015:52(2):331–340. 10.1111/1365-2664.12385 25954053 PMC4415536

[CIT0046] Mattila HR , Seeley TD. Genetic diversity in honey bee colonies enhances productivity and fitness. Science. 2007:317(5836):362–364. 10.1126/science.1143046 17641199

[CIT0047] Mondet F , Beaurepaire A, McAfee A, Locke B, Alaux C, Blanchard S, Danka B, Le Conte Y. Honey bee survival mechanisms against the parasite *Varroa destructor*: a systematic review of phenotypic and genomic research efforts. Int J Parasitol. 2020:50(6-7):433–447. 10.1016/j.ijpara.2020.03.005 32380096

[CIT0048] Morfin N , Goodwin PH, Guzman-Novoa E. Interaction of field realistic doses of clothianidin and *Varroa destructor* parasitism on adult honey bee (*Apis mellifera* L.) health and neural gene expression, and antagonistic effects on differentially expressed genes. PLoS One. 2020:15(2):e0229030. 10.1371/journal.pone.0229030 32078633 PMC7032720

[CIT0049] Mortensen AN , Jack CJ, Bustamante TA, Schmehl DR, Ellis JD. Effects of supplemental pollen feeding on honey bee (Hymenoptera: Apidae) colony strength and *Nosema* spp. infection. J Econ Entomol. 2019:112(1):60–66. 10.1093/jee/toy341 30388242

[CIT0050] Mullin CA , Frazier M, Frazier JL, Ashcraft S, Simonds R, vanEngelsdorp D, Pettis JS. High levels of miticides and agrochemicals in North American apiaries: implications for honey bee health. PLoS One. 2010:5(3):e9754. 10.1371/journal.pone.0009754 20333298 PMC2841636

[CIT0051] Oldroyd BP , Rinderer TE, Harbo JR, Buco SM. Effects of intracolonial genetic diversity on honey bee (Hymenoptera: Apidae) colony performance. Ann Entomol Soc Am. 1992:85(3):335–343. 10.1093/aesa/85.3.335

[CIT0052] Oldroyd BP , Thompson GJ. Behavioural genetics of the honey bee *Apis mellifera*. In: Simpson SJ, editor. Advances in insect physiology. Cambridge, Massachusetts: Academic Press; 2006. p. 1–49.

[CIT0053] Pettis JS , Delaplane KS. Coordinated responses to honey bee decline in the USA. Apidologie. 2010:41(3):256–263. 10.1051/apido/2010013

[CIT0054] Potts SG , Biesmeijer JC, Kremen C, Neumann P, Schweiger O, Kunin WE. Global pollinator declines: trends, impacts and drivers. Trends Ecol Evol. 2010:25(6):345–353. 10.1016/j.tree.2010.01.007 20188434

[CIT0055] Potts SG , Imperatriz-Fonseca V, Ngo HT, Aizen MA, Biesmeijer JC, Breeze TD, Dicks LV, Garibaldi LA, Hill R, Settele J, et al. Safeguarding pollinators and their values to human well-being. Nature. 2016:540(7632):220–229. 10.1038/nature20588 27894123

[CIT0056] R Core Team. R: A language and environment for statistical computing. Vienna, Austria: The R Foundation for Statistical Computing; 2019.

[CIT0057] Rangel J , Keller JJ, Tarpy DR. The effects of honey bee (*Apis mellifera* L.) queen reproductive potential on colony growth. Insect Soc. 2013:60(2):65–73.

[CIT0069] Straub L , Villamar-Bouza L, Bruckner S, Chantawannakul P, Gauthier L, Khongphinitbunjong K, Retschnig G, Troxler A, Vidondo B, Neumann P, et al. Neonicotinoid insecticides can serve as inadvertent insect contraceptives. Proc Biol Sci. 2016:283(1835):20160506. 10.1098/rspb.2016.0506 27466446 PMC4971197

[CIT0058] Roth MA , Wilson JM, Tignor KR, Gross AD. Biology and management of *Varroa destructor* (Mesostigmata: Varroidae) in *Apis mellifera* (Hymenoptera: Apidae) colonies. J Integr Pest Manag. 2020:11(1):1.

[CIT0059] Sammataro D , Weiss M. Comparison of productivity of colonies of honey bees, *Apis mellifera*, supplemented with sucrose or high fructose corn syrup. J Insect Sci. 2013:13(19):1–13. 10.1673/031.013.1901 23886010 PMC3735052

[CIT0060] Sánchez-Bayo F , Goulson D, Pennacchio F, Nazzi F, Goka K, Desneux N. Are bee diseases linked to pesticides? A brief review. Environ Int. 2016:89-90(9–90):7–11. 10.1016/j.envint.2016.01.009 26826357

[CIT0082] Seeley TD . Adaptive significance of the age polyethism schedule in honeybee colonies. Behavioral Ecol Sociobio. 1982;11:287–293.

[CIT0061] Seeley TD , Tarpy DR. Queen promiscuity lowers disease within honeybee colonies. Proc Biol Sci. 2007:274(1606):67–72. 10.1098/rspb.2006.3702 17015336 PMC1679871

[CIT0062] Sherman PW , Seeley TD, Reeve HK. Parasites, pathogens, and polyandry in social hymenoptera. Am Naturalist. 1988:131(4):602–610. 10.1086/284809 18811329

[CIT0063] Shykoff JA , Schmid-Hempel P. Genetic relatedness and eusociality: parasite-mediated selection on the genetic composition of groups. Behav Ecol Sociobiol. 1991a:28:371–376.

[CIT0064] Shykoff JA , Schmid-Hempel P. Parasites and the advantage of genetic variability within social insect colonies. Proc R Soc Lond Ser B Biol Sci. 1991b:243(1306):55–58.

[CIT0065] Siefert P , Hota R, Ramesh V, Grünewald B. Chronic within-hive video recordings detect altered nursing behaviour and retarded larval development of neonicotinoid treated honey bees. Sci Rep. 2020:10(1):8727. 10.1038/s41598-020-65425-y 32457387 PMC7251098

[CIT0066] Singla A , Barmota H, Kumar Sahoo S, Kaur Kang B. Influence of neonicotinoids on pollinators: a review. J Api Res. 2021:60(1):19–32. 10.1080/00218839.2020.1825044

[CIT0067] Singmann H , Bolker B, Westfall J, Aust F, Ben-Shachar MS. afex: analysis of factorial experiments. R Package; 2019.

[CIT0068] Siviter H , Bailes EJ, Martin CD, Oliver TR, Koricheva J, Leadbeater E, Brown, MJF. Agrochemicals interact synergistically to increase bee mortality. Nature. 2021:596(8):1–4. 10.1038/s41586-021-03787-7 34349259

[CIT0081] Rinderer TE , Collins AM, Pesante D, Daniel R, Lancaster V, Baxter J. A comparison of Africanized and European drones: weights, mucus gland and seminal vesicle weights, and counts of spermatozoa. Apidologie. 1985;16(4):407–412.

[CIT0070] Straub L , Villamar-Bouza L, Bruckner S, Chantawannakul P, Kolari E, Maitip J, Vidondo B, Neumann P, Williams GR. Negative effects of neonicotinoids on male honeybee survival, behaviour and physiology in the field. J Appl Ecol. 2021:58(11):2515–2528.

[CIT0071] Straub L , Williams GR, Vidondo B, Khongphinitbunjong K, Retschnig G, Schneeberger A, Chantawannakul P, Dietemann V, Neumann P. Neonicotinoids and ectoparasitic mites synergistically impact honeybees. Sci Rep. 2019:9(1):8159. 10.1038/s41598-019-44207-1 31164662 PMC6547850

[CIT0072] Tarpy DR , Nielsen R, Nielsen DI. A scientific note on the revised estimates of effective paternity frequency in Apis. Insect Soc. 2004:51(5):203–204.

[CIT0073] Tarpy DR , Seeley TD. Lower disease infections in honeybee (*Apis mellifera*) colonies headed by polyandrous vs monandrous queens. Naturwissenschaften. 2006:93(4):195–199. 10.1007/s00114-006-0091-4 16518641

[CIT0074] Tarpy DR , vanEngelsdorp D, Pettis JS. Genetic diversity affects colony survivorship in commercial honey bee colonies. Naturwissenschaften. 2013:100(8):723–728. 10.1007/s00114-013-1065-y 23728203

[CIT0075] Tesovnik T , Zorc M, Gregorc A, Rinehart T, Adamczyk J, Narat M. Immune gene expression in developing honey bees (*Apis mellifera* L.) simultaneously exposed to imidacloprid and *Varroa destructor* in laboratory conditions. J Apic Res. 2019:58(5):730–739. 10.1080/00218839.2019.1634463

[CIT0076] Traynor KS , Mondet F, de Miranda JR, Techer M, Kowallik V, Oddie MAY, Chantawannakul P, McAfee A. *Varroa destructor*: a complex parasite, crippling honey bees worldwide. Trends Parasitol. 2020:36(7):592–606. 10.1016/j.pt.2020.04.004 32456963

[CIT0077] van Alphen JJ , Fernhout BJ. Natural selection, selective breeding, and the evolution of resistance of honeybees (*Apis mellifera*) against Varroa. Zool Lett. 2020:6:1–20. 10.1186/s40851-020-00158-4 PMC7236208 32467772

[CIT0078] vanEngelsdorp D , Meixner MD. A historical review of managed honey bee populations in Europe and the United States and the factors that may affect them. J Invertebr Pathol. 2010:103(Suppl 1):S80–S95. 10.1016/j.jip.2009.06.011 19909973

[CIT0079] Withrow JM , Tarpy DR. Cryptic ‘royal’ subfamilies in honey bee (*Apis mellifera*) colonies. PLoS One. 2018:13(7):e0199124. 10.1371/journal.pone.0199124 29995879 PMC6040692

[CIT0080] Zee R. van der, Gray A, Pisa L, Rijk T. de. An observational study of honey bee colony winter losses and their association with *Varroa destructor*, neonicotinoids and other risk factors. PLoS One. 2015:10(7):e0131611. 26154346 10.1371/journal.pone.0131611 PMC4496033

