# Supplementary material for: Neonicotinoid exposure increases Varroa destructor (Mesostigmata: Varroidae) mite parasitism severity in honey bee colonies and is not mitigated by increased colony genetic diversity
Source: J Insect Sci. 2024 May 28;24(3):20. doi: 10.1093/jisesa/ieae056 (PMC11132139; doi:10.1093/jisesa/ieae056)
Supplement: ieae056_suppl_Supplementary_Material [file ieae056_suppl_supplementary_material.docx]

**Supplementary Material***Neonicotinoid exposure increases Varroa parasitism severity in honey bee colonies and is not mitigated by increased colony genetic diversity.*

Lewis J. Bartlett^1,4,^†, Suleyman Alparslan^2^, Selina Bruckner^2^, Deborah A. Delaney^3^, John F. Menz^3^, Geoffrey R. Williams^2^, Keith S. Delaplane^4,^*

^1^Center for the Ecology of Infectious Diseases, University of Georgia, Athens, GA 30602 USA
ORCID (LJB): 0000-0002-4418-8071

† lewis.bartlett@uga.edu

^2^Department of Entomology & Plant Pathology, Auburn University, Auburn, AL 36849 USA

ORCID (SA): NA
ORCID (SB): NA
ORCID (GRW): 0000-0002-0093-1126

^3^Department of Entomology & Wildlife Ecology, University of Delaware, Newark, DE 27695-7613 USA

ORCID (JFM): NA

ORCID (DAD): 0000-0001-7715-8218

^4^Department of Entomology, University of Georgia, Athens, GA 30602 USA
ORCID (KSD): 0000-0001-9323-441X
*ksd@uga.edu


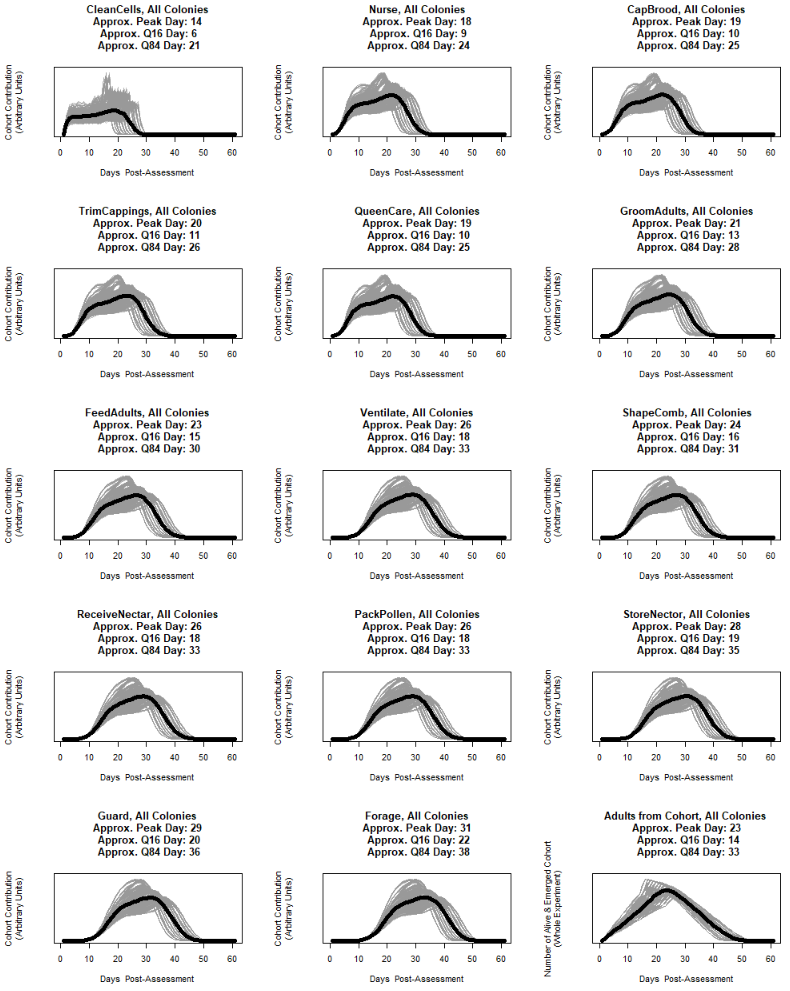


**Figure S1**  - Task emphasis graphs for the Alabama site experiment, used to target sampling windows for more intensive sampling protocols. Grey shading represents uncertainty based on stochastic simulation outcomes, unknown exact ages of brood, and discrepancies in agreement between colony assessors. The Alabama site had more eggs and young brood than other sites, with later and ‘flatter’ task emphasis prediction curves.


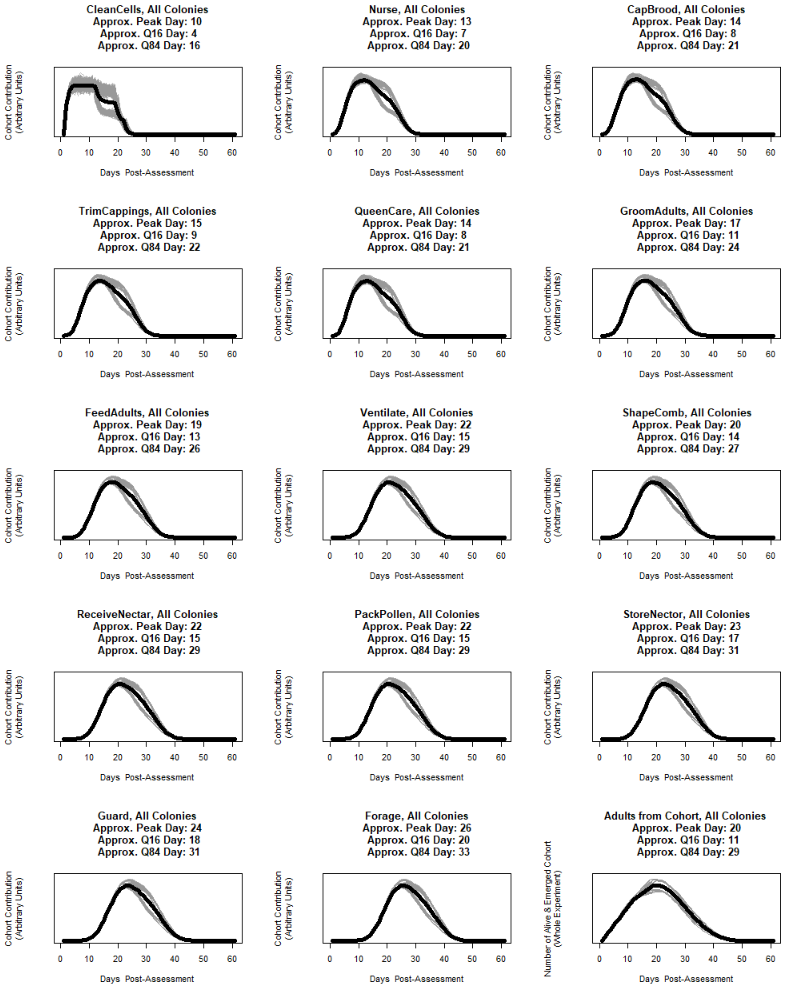


**Figure S2**  - Task emphasis graphs for the Delaware site experiment, used to target sampling windows for more intensive sampling protocols. Grey shading represents uncertainty based on stochastic simulation outcomes, unknown exact ages of brood, and discrepancies in agreement between colony assessors. The Delaware site was dominated by capped brood compared to other sites, with sooner and ‘sharper’ task emphasis prediction curves.


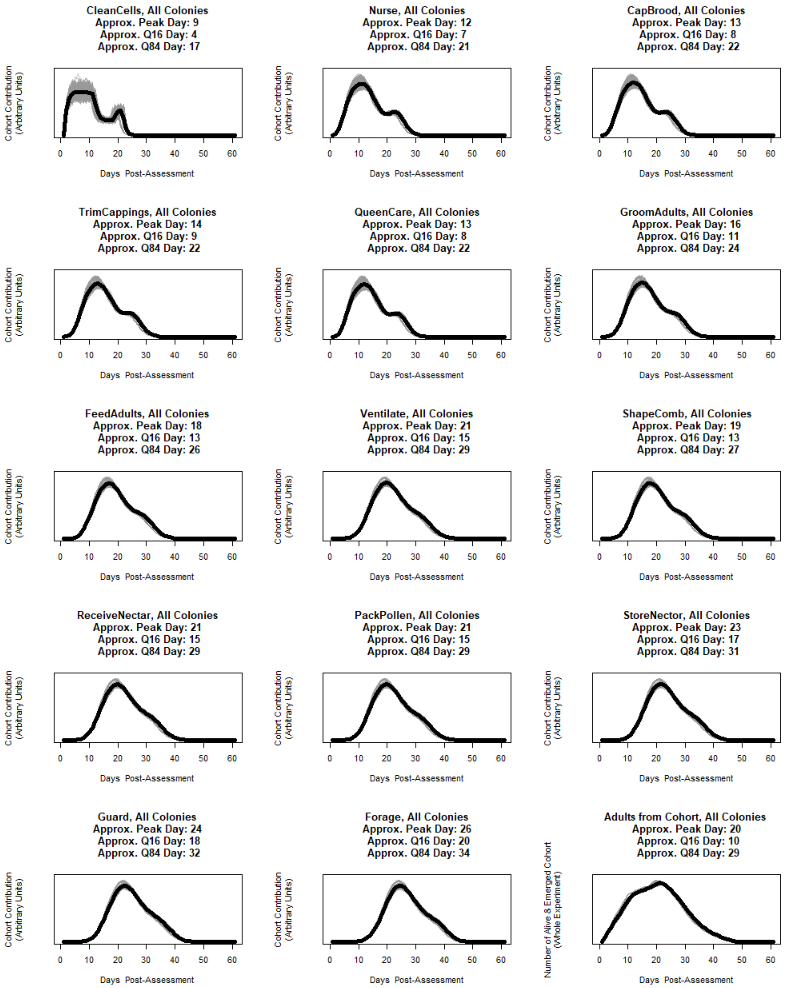


**Figure S3**  - Task emphasis graphs for the Georgia site experiment, used to target sampling windows for more intensive sampling protocols. Grey shading represents uncertainty based on stochastic simulation outcomes, unknown exact ages of brood, and discrepancies in agreement between colony assessors. The Georgia site had a majority of capped brood and a secondary large minority of eggs compared to other sites, leading to a ‘trailing delay’ in task emphasis peaks, with a smaller secondary peak later than the first.
